# Supplementary figures and images for: A novel monomeric amyloid β-activated signaling pathway regulates brain development via inhibition of microglia
Source: eLife. 2024 Dec 5;13:RP100446. doi: 10.7554/eLife.100446 (PMC11620749; doi:10.7554/eLife.100446)

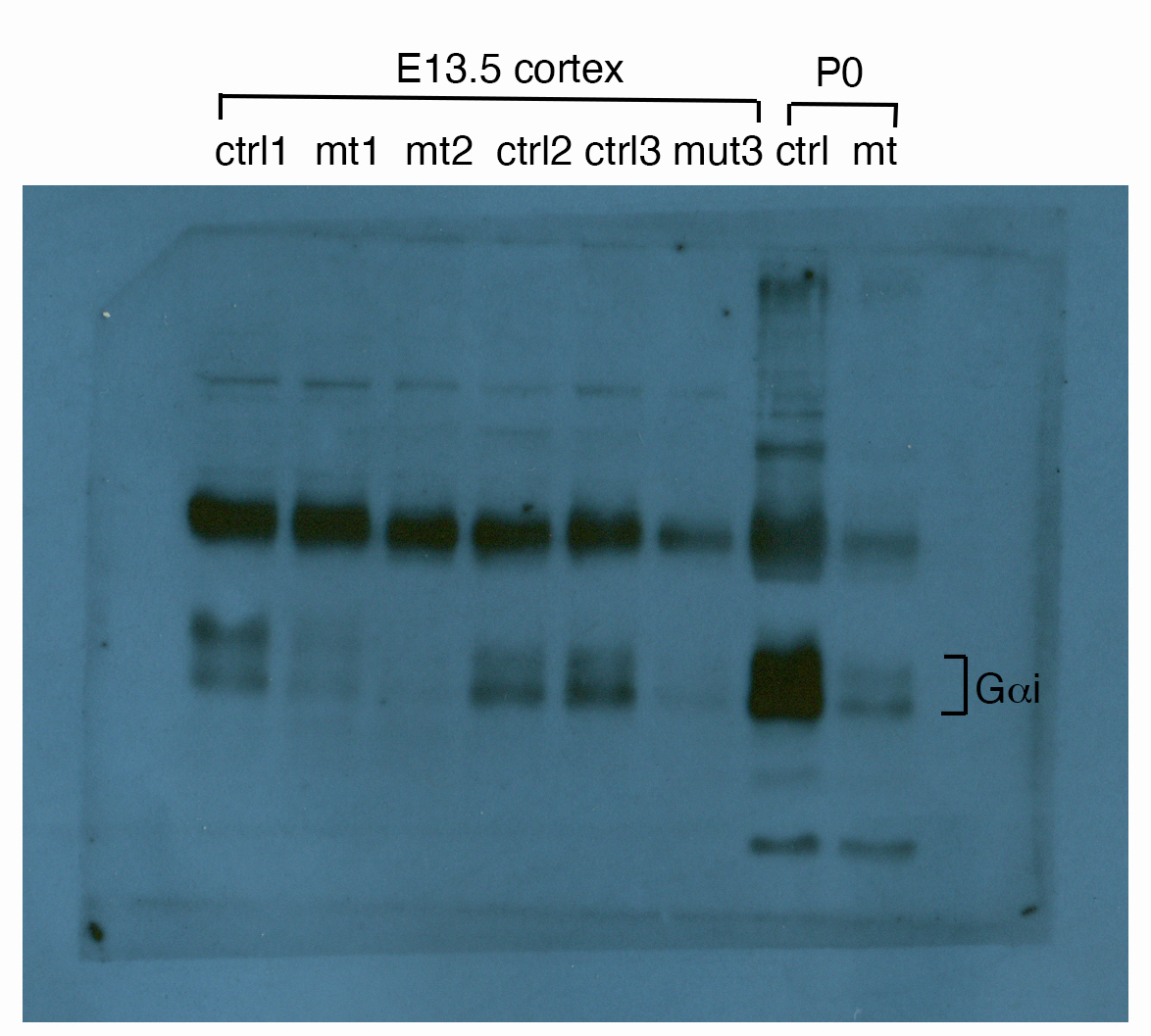

Supplement: Figure 2—figure supplement 2—source data 1. [file elife-100446-fig2-figsupp2-data1.zip › Figure 2-figure supplement 2 source data 1/Gi Western labeled data.jpg]

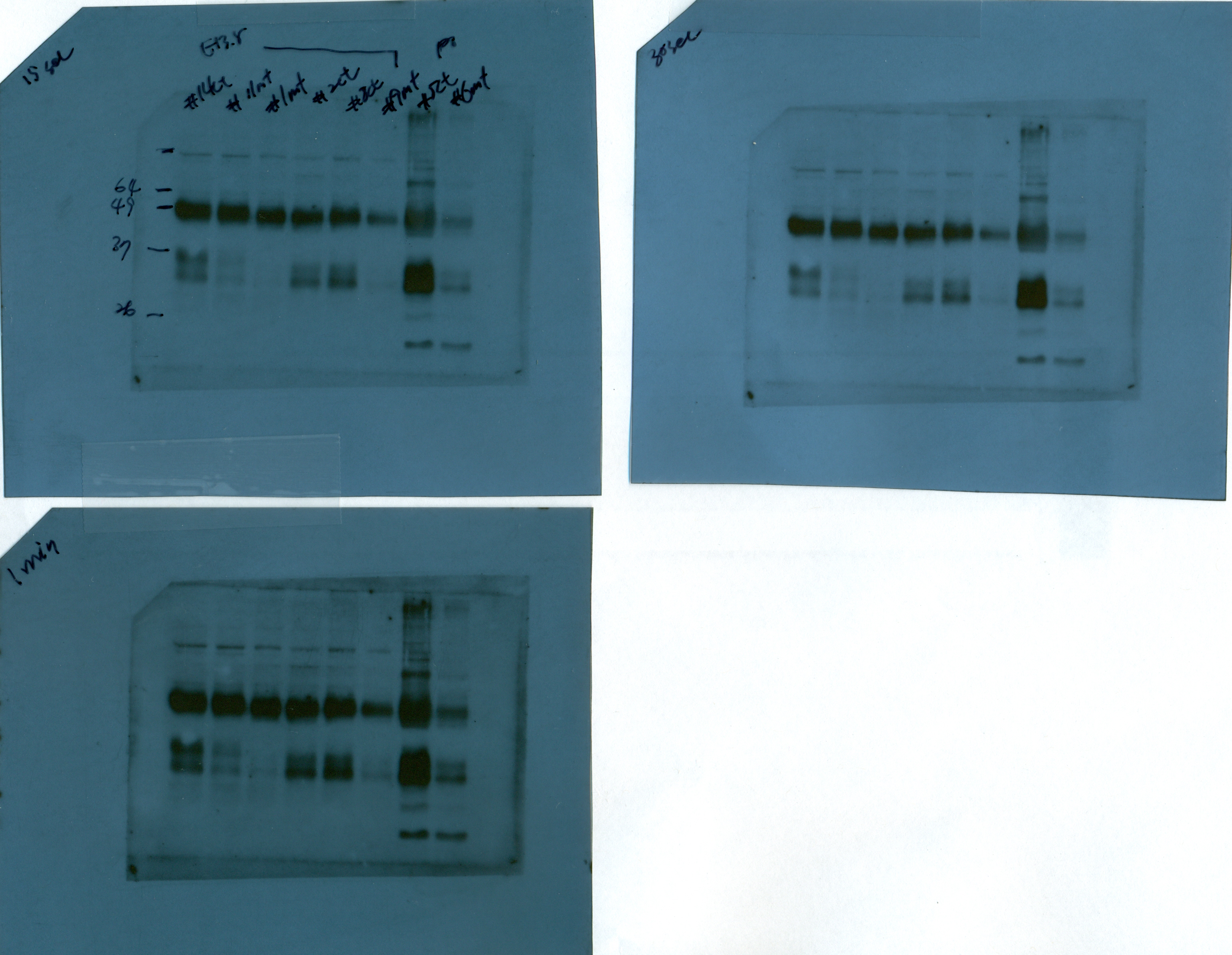

Supplement: Figure 2—figure supplement 2—source data 2. [file elife-100446-fig2-figsupp2-data2.zip › Figure 2-figure supplement 2 source data 2/Galpha i western blot 3pairs e13.5 and one pair P0 2-3-12.jpg]

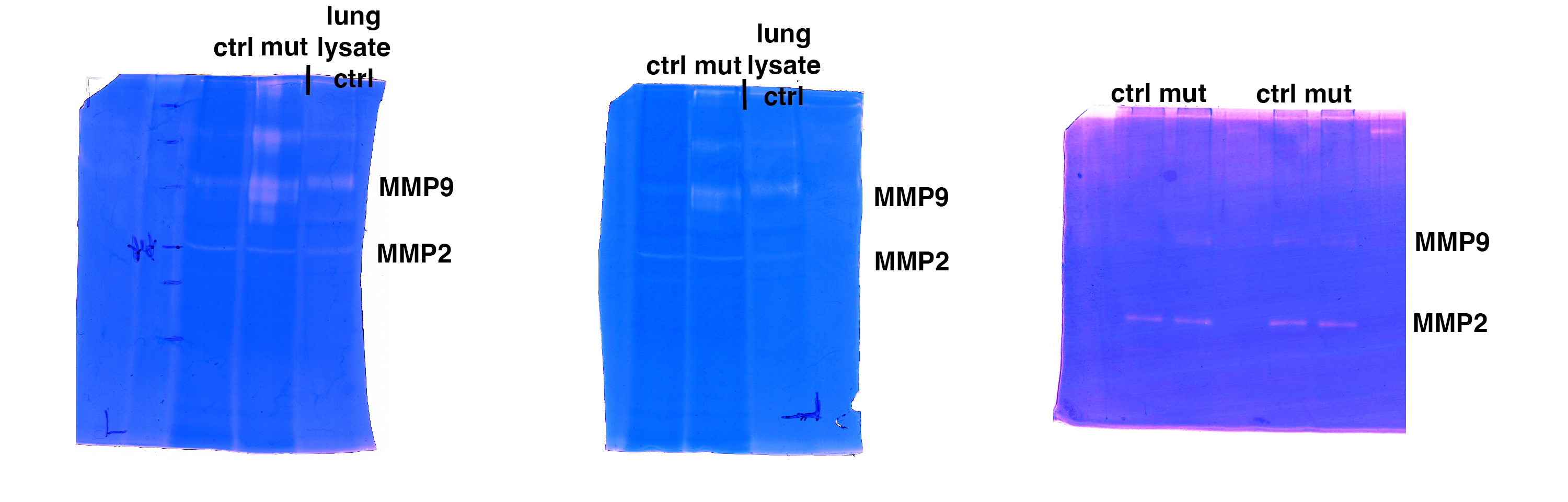

Supplement: Figure 5—figure supplement 2—source data 1. — Embryonic lung lysates were used as control for validation of MMP2/9 activity. [file elife-100446-fig5-figsupp2-data1.zip › Figure 5-figure supplement 2 source data 1/Zymography labeled.jpg]

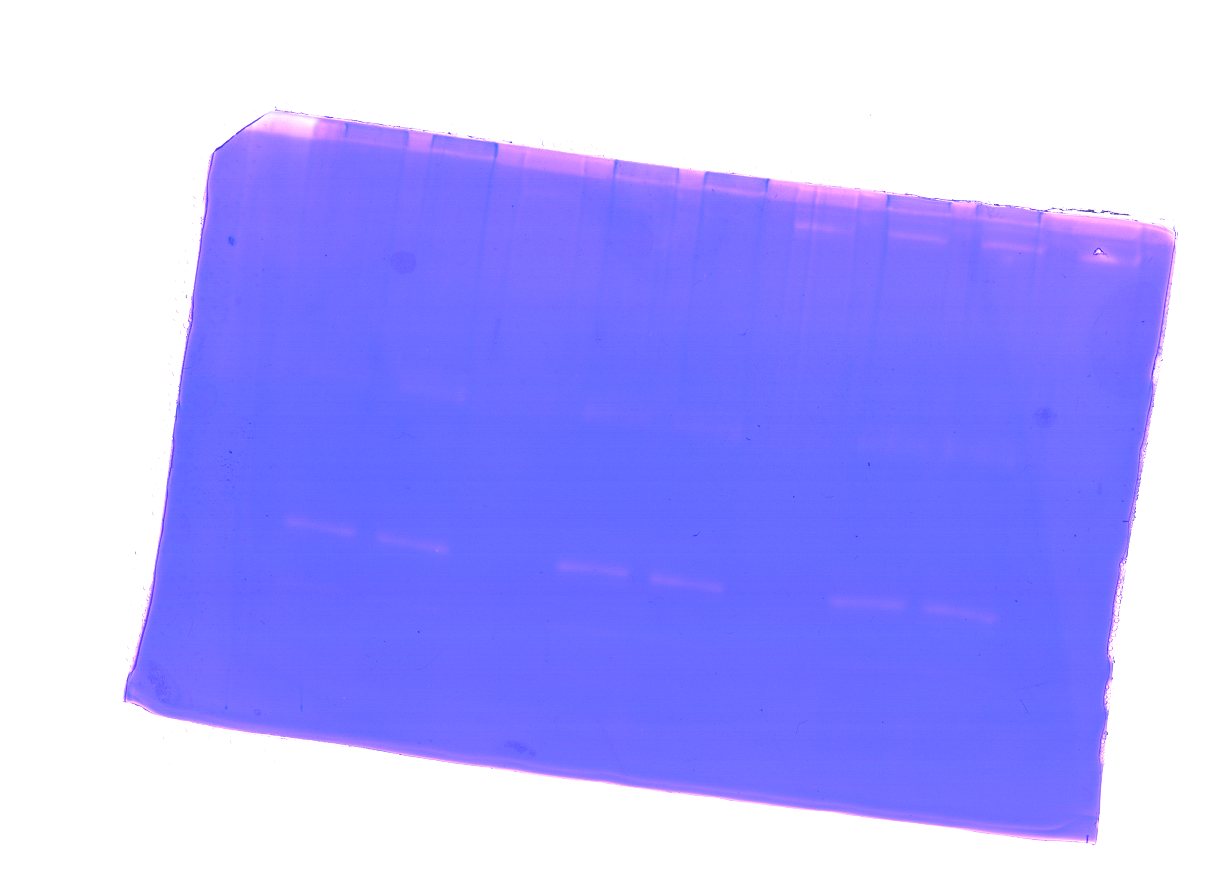

Supplement: Figure 5—figure supplement 2—source data 2. [file elife-100446-fig5-figsupp2-data2.zip › Figure 5-figure supplement 2 source data 2/zymography 9-2-10.jpg]

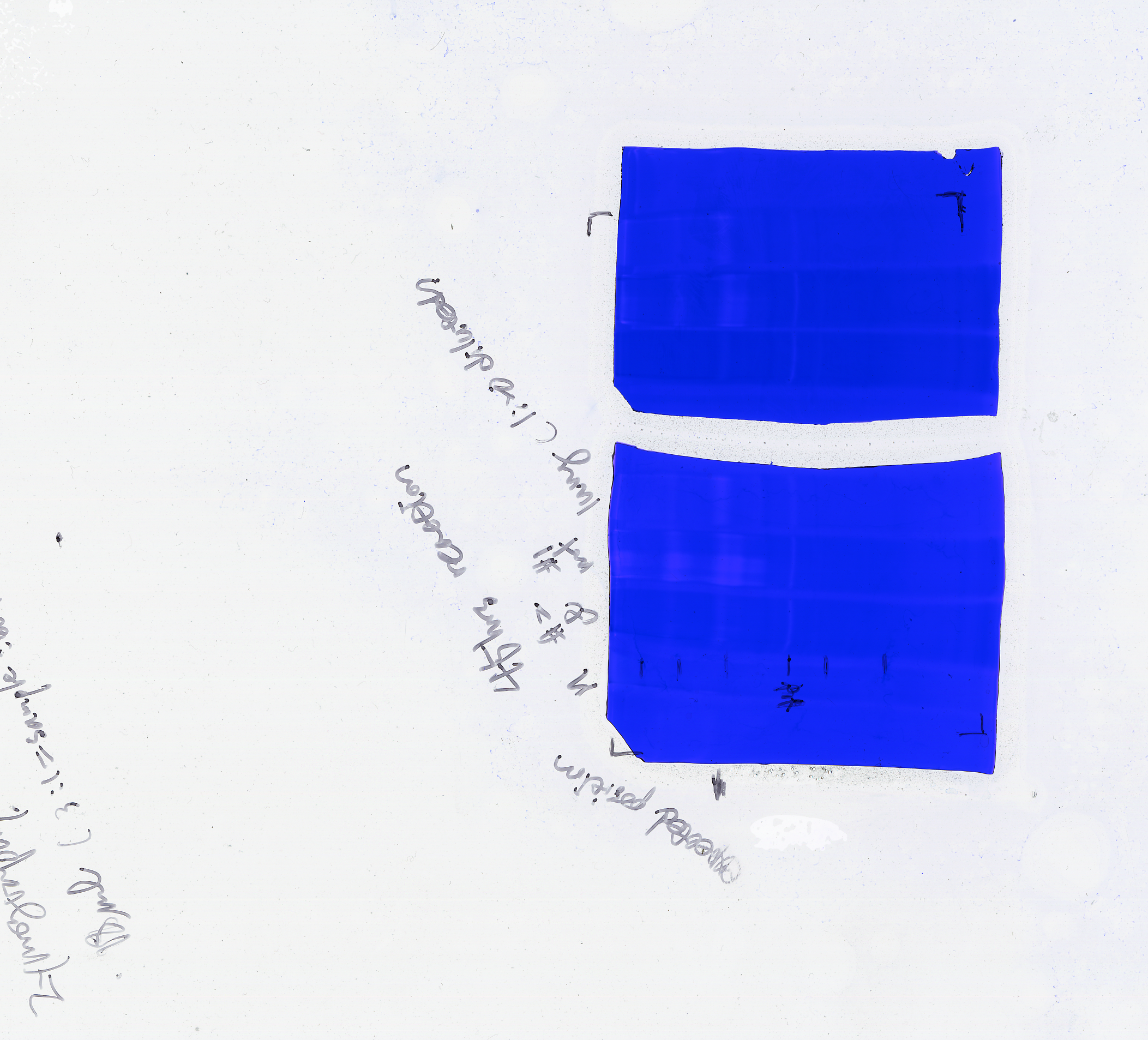

Supplement: Figure 5—figure supplement 2—source data 2. [file elife-100446-fig5-figsupp2-data2.zip › Figure 5-figure supplement 2 source data 2/zymography 12-28-11.tif]
